# Atomically Thin 2D Multinary Nanosheets for Energy‐Related Photo, Electrocatalysis

**DOI:** 10.1002/advs.201800244

**Published:** 2018-05-07

**Authors:** Jun Xiong, Jun Di, Huaming Li

**Affiliations:** ^1^ School of Chemistry and Chemical Engineering Institute for Energy Research Jiangsu University 301 Xuefu Road Zhenjiang 212013 P. R. China; ^2^ School of Mechanical and Aerospace Engineering Nanyang Technological University Singapore 639798 Singapore; ^3^ School of Materials Science and Engineering Nanyang Technological University Singapore 639798 Singapore

**Keywords:** atomically thin nanosheets, electrocatalytic processes, multinary nanosheets, photocatalytic processes, sustainable energy

## Abstract

The severe energy crisis and environmental issues have led to an increase in research on the development of sustainable energy. Atomically thin 2D multinary nanosheets with tunable components show advantages for producing sustainable energy via photo, electrocatalysis processes. Here, recent progress of atomically thin 2D multinary nanosheets from the design, synthesis, tuning, and sustainable energy production via photo, electrocatalysis processes is summarized. The regulating strategies such as alloying, doping, vacancy engineering, pores construction, surface modification, and heterojunction are summarized, focusing on how to optimize the catalytic performance of atomically thin 2D multinary nanosheets. In addition, advancements in versatile energy‐related photo, electrocatalytic applications in the areas of oxygen evolution, oxygen reduction, hydrogen evolution, CO_2_ reduction, and nitrogen fixation are discussed. Finally, existing challenges and future research directions in this promising field are presented.

## 1 Introduction

With the drastic consumption of fossil fuels, global energy shortages and environmental pollution problems are increasingly aggravation.^1^, ^2^ It is highly desirable to develop sustainable, fossil‐free pathways to produce renewable fuels. Among numerous approaches, splitting water to produce hydrogen, reducing CO_2_ to carbon‐based fuels via photo, electrocatalysis processes are regarded as perspective strategies for future energy sustainability.^3^, ^4^, ^5^ Molecular hydrogen have the advantages of high specific energy, multiple utilization modes, and clean combustion product, which is desired fuel to replace fossil fuels. On the other side, by virtue of renewable sources, converting CO_2_ to useful hydrocarbon fuels and chemicals provides a highly desirable solution to artificial carbon recycling.^6^ The critical requirement for realizing a high‐efficiency catalytic conversion process is the reasonable design of photo, electrocatalysts, which has attracted substantial research interest. Morphology structure of photo, electrocatalysts is one of the important factors to affect the catalytic performance.

Atomically thin 2D nanosheets (including both layered and nonlayered structures) with only single‐ or few‐atoms thick (typically less than 5 nm) represent an emerging class of nanomaterials that attracted significant attention for photo, electrocatalysis to produce sustainable energy.^7^, ^8^ Benefiting from the distinctive 2D morphology and ultrathin thickness, the atomically thin 2D nanosheets manifest several extraordinary physical, chemical and electronic features relative to the corresponding bulk counterpart, and thus display inspirational potential for applications such as electronics, catalysis and energy storage.^9^, ^10^, ^11^ With respect to photo, electrocatalytic process, the atomically thin 2D nanosheets have several unique advantages.^12^ First of all, compared to their bulk counterparts, atomically thin 2D nanosheets display larger surface‐to‐volume ratio, enabling more target molecule adsorbed on the surface. For another, the atomic thickness is of great benefit to light absorption and can greatly shorten the charge diffusion distance from the interior to the surface for photocatalysis. The ultra‐flexible features of atomically thin 2D nanosheets can maximize the electron accessibility to the electrode substrate and build an intimate contact in electrocatalysis, so as to lower interfacial electron transfer resistance. The high in‐plane electron transport mobility of 2D layered structure allows the fast electron migration and favors the electrocatalytic process. Moreover, the abundant coordination‐unsaturated surface atoms in atomically thin 2D nanosheets can provide more active sites to involve in the interfacial catalytic reactions.^13^, ^14^ In addition, when the thickness of materials is reduced to single or few layers, the strong quantum confinement effect will bring about novel electronic and optical properties. For example, the indirect type bulk MoS_2_ with bandgap of 1.3 eV can hardly proceed photocatalytic reactions, while the single layer MoS_2_ with bandgap of ≈1.84 eV change to direct type and provide the appropriate band structure for photocatalytic process. The similar phenomenon can also be observed in black phosphorous with bandgap increase from 0.3 eV for bulk sample to 2.1 eV for monolayer sample.^14^ As a consequence, the atomically thin 2D nanosheets are excellent platforms to perform the catalytic process for energy conversion.

Another important factor to affect the catalytic performance is the component of materials. Reasonable tune the component of the materials can effectively adjust its electronic structure and surface properties of catalysts, and thus further optimize the catalytic performance.^15^ The widely studied 2D nanosheets in photo, electrocatalytic process such as g‐C_3_N_4_ and MoS_2_ are usually binary, which limit its further component tuning and performance optimizing. Recent studies found that constructing 2D multinary nanosheets is a compelling strategy to further adjust their properties such as optical, electronic and chemical properties and finally optimize their performance.^16^ For example, alloyed 2D BCN materials can display outstanding photocatalytic hydrogen evolution and CO_2_ photoreduction behavior, while the binary BN has been regarded as insulator without photocatalytic activity.^17^ These 2D multinary nanosheets displayed some unique advantages compared to 2D binary materials, endowing them to be fundamentally important in photo, electrocatalytic reactions.

Herein, we present a comprehensive overview of the latest research progress in atomically thin 2D multinary nanosheets toward photo, electrocatalysis to produce sustainable energy. We start with a synthetic method of preparing new atomically thin 2D multinary photo, electrocatalysts. Then, we discussed the regulating approaches for tuning the catalytic performance of atomically thin 2D multinary nanosheets, such as alloying, doping, vacancy engineering, pores construction, surface modification and heterojunction. After that, the different energy catalytic applications over atomically thin 2D multinary nanosheets are introduced. Finally, conclusions and a forward‐looking outlook of these emerging atomically thin 2D multinary nanosheets are also discussed.

## 2 Synthesis of Atomically Thin 2D Multinary Photo, Electrocatalysts

Atomically thin 2D multinary nanosheets for photoelectric energy catalysis can be mainly categorized as metal chalcogenide, oxyhalide, oxometallate, hydroxides, metal‐organic framework (MOFs), and others. Both metal or chalcogen components can be tuned in multinary metal chalcogenide, such as ZnIn_2_S_4_, NiCo_2_S_4_, MoSeS, and MoS_2(1−_ *_x_*_)_Se_2_ *_x_*.^18^, ^19^, ^20^, ^21^, ^22^, ^23^, ^24^, ^25^ The oxyhalide main focused on bismuth oxyhalides with adjustable halogen types and oxygen/halogen ratio, such as BiOCl, BiOBr, BiOI, Bi_12_O_17_Cl_2_, Bi_3_O_4_Br, Bi_4_O_5_Br_2_, and so on.^26^, ^27^, ^28^, ^29^, ^30^, ^31^ The oxometallate mainly includes Ca_2_Nb_3_O_10_, Ba_5_Nb_4_O_15_, SnNb_2_O_6_, K_4_Nb_6_O_17_, SrNb_2_O_6_, HNbWO_6_, Bi_2_WO_6_, Bi_2_MoO_6_, and BiVO_4_.^32^, ^33^, ^34^, ^35^, ^36^, ^37^, ^38^, ^39^, ^40^, ^41^, ^42^, ^43^ The hydroxides can be classified as single metal hydroxides (e.g., Ni(OH)_2_) and layered double hydroxides (LDHs) including CoCo LDHs, NiCo LDHs, NiFe LDHs, Ni_0.75_V_0.25_ LDHs, CoMn LDHs, CoFe LDHs, NiCoFe LDHs, ZnAl LDHs, CuCr LDHs, and MgAl LDHs.^44^, ^45^, ^46^, ^47^, ^48^, ^49^, ^50^, ^51^, ^52^, ^53^ In addition, the MOFs with the tuned metal centers can also serve as effective catalysts, such as NiCo MOFs,^54^ NiFe‐MOFs.^55^

**Figure 1:**
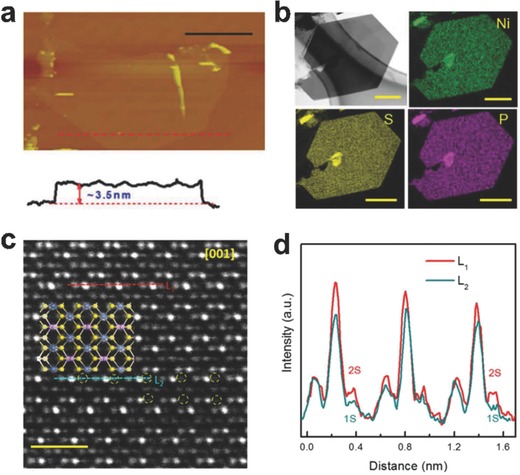
a) AFM images and the corresponding height analysis of the hexagonal few layered and multilayered NiPS_3_ crystals, scale bar 5 µm. b) The EDX elemental mapping of Ni, S, and P cross the hexagonal sheet, scale bar 500 nm. c) Atomic‐level HAADF‐STEM image of an ultrathin NiPS_3_ nanosheet showing the sulfur vacancies (yellow circles) and the corresponding structural schematic. FFT mask filter has been employed for clarity, scale bar 1 nm. d) Intensity profiles along lines L1 and L2. Higher contract is obtained from the Ni atom compared to S atom. Reproduced with permission.^56^ Copyright 2017, Elsevier.

**Figure 2:**
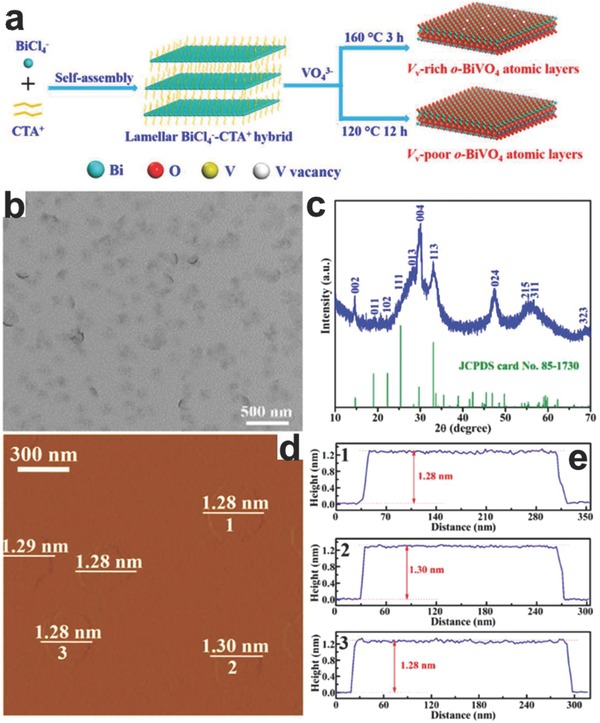
Scheme and characterizations for the synthetic o‐BiVO_4_ atomic layers. a) Scheme for the gram‐scale synthesis of the V_v_‐rich and V_v_‐poor o‐BiVO_4_ atomic layers. Characterizations for the V_v_‐rich o‐BiVO_4_ atomic layers with one‐unit‐cell thickness: b) TEM image, c) XRD pattern, d) AFM image, and e) the corresponding height profiles; the numbers from 1 to 3 in (d) correspond to the numbers from 1 to 3 in (e). Reproduced with permission.^43^ Copyright 2017, the American Chemical Society.

Generally, the atomically thin 2D multinary photo, electrocatalysts can be prepared via several methods, such as chemical vapor deposition (CVD), sonication‐assisted liquid exfoliation, chemical Li intercalation‐assisted exfoliation, surfactant self‐assembly method and inorganic–organic lamellar hybrid intermediate strategy. For instance, He's group developed a CVD process to grow few‐atomic layered metal phosphorus trichalcogenides NiPS_3_ (**Figure** **1**).^56^ By virtue of beforehand prepared ultrathin Ni(OH)_2_, the NiPS_3_ nanosheets with 3.5 nm thick can be synthesized through reaction with sulfur and phosphorus at high temperature. The obtained NiPS_3_ nanosheets can decompose pure water to produce hydrogen without any cocatalysts or sacrificial agents under simulated solar light irradiation. However, no stoichiometric oxygen can be released in this system due to the insufficient intrinsic driving force of valence band (VB) and the oxidation products need to be further explored. Due to the harsh synthesize requirement and low catalyst yield, this method may not very suitable for photo, electrocatalysis. Similar to the synthetic process of binary atomically thin 2D nanosheets with layered structures, the sonication‐assisted liquid exfoliation is also an effective pathway to prepare atomically thin 2D multinary nanosheets with van der Waals layered structures. Hu's group demonstrated the preparation of CoCo, NiCo, and NiFe LDH nanosheets with the thickness of single layer via the sonication‐assisted liquid exfoliation process from corresponding bulk LDHs.^46^ The exfoliated single layer structured LDHs displayed greatly improved activity than the corresponding bulk counterparts toward electrocatalytic oxygen evolution reaction (OER) in alkaline conditions. Although the sonication‐assisted liquid exfoliation method has the advantages such as ease of control, economic and higher yield than CVD process, it still suffer from the shortage of wide range of thickness for products and relatively low exfoliation efficiency, especially for the single‐layer nanosheets. To improve the productivity of single‐layer nanosheets, chemical Li intercalation‐assisted exfoliation may be an effective strategy.^57^, ^58^ By intercalating ions into the interlayer of layered materials, the interlayer distances will be enlarged and interlayered van der Waals interaction will be weakened. As a result, a more efficient process can be achieved to prepare single‐ or few‐layer nanosheets by ultrasound. Zhang's group synthesized the Bi_12_O_17_Cl_2_ monolayers via a chemical Li intercalation‐assisted exfoliation process by intercalating lithium into the spaces between each neighboring Bi_12_O_17_Cl_2_ monolayer‐unit under argon atmosphere for 72 h at 100 °C.^29^ After further ultrasonic treatment, the thickness of the obtained Bi_12_O_17_Cl_2_ nanosheets was determined to be 0.717 nm by atomic force microscopy (AFM), well‐matching with the theoretical value of Bi_12_O_17_Cl_2_ monolayers. It should be noted that the phase transition may happen during the Li intercalation‐assisted exfoliation approach. However, the exfoliation strategy request intrinsic layered structure of bulk materials with interlayer van der Waals interaction. With respect to the nonlayered structured 2D multinary nanomaterials, the lack of intrinsic anisotropic growth driving force and strong binding energy greatly limit the use of exfoliation method. To address this issue, surfactant self‐assembly method was developed to prepare different atomically thin 2D multinary nanosheets directly.^59^, ^60^, ^61^ Our group employed polyvinyl pyrrolidone (PVP) as surfactant to control the formation of atomically thin BiOCl nanosheets via a solvothermal process.^26^ The PVP can prevent the further growth of BiOCl nanocrystals by creating a passivation layer around BiOCl cores. By virtue of the repulsive forces among the polyvinyl groups, the growth of BiOCl along its *c*‐axis is effective suppressed and an ultrathin structure is achieved. Compared to the bulk BiOCl, the ultrathin structured BiOCl displayed greatly increased photocatalytic oxygen evolution activity. In addition, Xie's group recently developed an inorganic–organic lamellar hybrid intermediate strategy to prepare the atomically thin 2D nanosheets and this strategy was also efficient for the multinary materials.^39^, ^43^ For instance, artificial lamellar BiCl_4_^−^‐CTA^+^ hybrid precursors was formed via the self‐assembly process by BiCl_3_ and cetyltrimethylammonium bromide, as certified by small‐angle XRD pattern.^43^ Subsequently, VO_4_^3−^ ions would react with the Bi^3+^ in the lamellar BiCl_4_^−^‐CTA^+^ hybrid to form BiVO_4_‐based lamellar structures during the hydrothermal process. Finally, it gradually self‐exfoliated into o‐BiVO_4_ atomic layers with one‐unit‐cell thickness (**Figure** **2**). The as‐prepared o‐BiVO_4_ atomic layers with rich vanadium vacancies exhibited outstanding photocatalytic methanol formation rate, arriving 398.3 µmol g^−1^ h^−1^. This is the highest value for methanol formation via photoreduction process over atomically thin 2D multinary nanosheets up to now.

## 3 Tuning Strategies for the Optimization of Catalytic Performance

In order to further improve the catalytic performance of atomically thin 2D multinary nanosheets, several strategies have been successfully employed such as alloying, doping, vacancy engineering, pores construction, surface modification and heterojunction. As such, the chemical, electronic, optical properties can be effective tuned and then optimize the catalytic activity toward sustainable energy production.

### 3.1 Alloying and Doping

**Figure 3:**
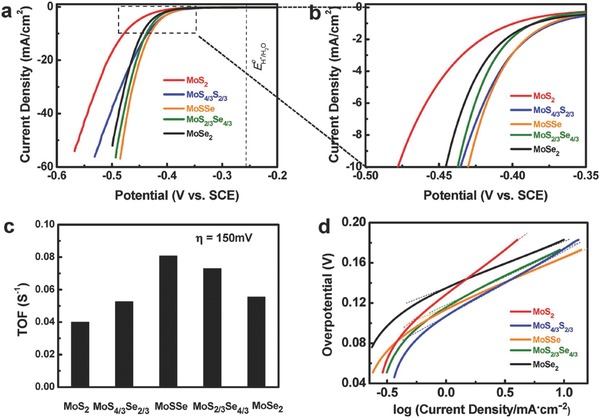
Electrochemical measurements of MoS_2(1−_ *_x_*_)_Se_2_ *_x_* nanoflakes for HER electrocatalysis in 0.5 m H_2_SO_4_: a,b) polarization curves, where the enclosed area of (a) is magnified in (b); c) turnover frequency at η = 150 mV estimated from CV curves; d) corresponding Tafel plots. Alloyed nanoflakes, particularly MoSSe, exhibit improved performance in comparison to either MoS_2_ or MoSe_2_. The vertical dotted line in (a) indicates the theoretical potential for HER. Reproduced with permission.^24^ Copyright 2017, the American Chemical Society.

Electronic structure of the semiconductors plays significant effect on the catalytic behavior. Alloying and doping have been regarded as efficient means to tune the electronic structure of materials.^62^, ^63^ Gong et al. synthesized MoS_2(1−_ *_x_*_)_Se_2_ *_x_* alloy nanoflakes with monolayer or few‐layer thickness and employed for electrocatalytic hydrogen evolution reaction.^24^ The optimized MoSSe displayed increased performance relative to either binary MoS_2_ or MoSe_2_, with η = 164 mV to achieve a current density of 10 mA cm^−2^ and turnover frequency (TOF) value arrive 0.0808 s^−1^ at η = 154 mV (**Figure** **3**). It is demonstrated that the alloyed Se modulates the d band electronic structure of Mo, thus further adjust hydrogen adsorption free energy and finally optimize electrocatalytic activity. Apart from catalytic activity improvement, rational alloying can even bring about novel property. By alloying C into BN to form ultrathin BCN ternary alloys, the electronic structure can be effective tuned.^17^ Different from the insulator h‐BN, the BCN ternary alloys possess a delocalized 2D electron system and a greatly decreased bandgap. As a consequence, the optimized BCN ternary alloys displayed photocatalytic hydrogen evolution and CO_2_ reduction performance under visible light irradiation, while the h‐BN do not has photocatalytic activity in fact.

In addition to alloying, doping is an universal effective means to promote the catalytic behavior.^64^ Yang et al. demonstrated the preparation of oxygen‐doped ZnIn_2_S_4_ nanosheets with five Zn–In–S molecular layers.^19^ Compared with the pristine ZnIn_2_S_4_ nanosheets, the oxygen substitution for lattice sulfur atoms in O‐doped ZnIn_2_S_4_ can increase the density of states (DOS) at VB maximum, result in the decrease of bandgap, upshifting of both VB and conduction band (CB) edges. Apart from the effect on electronic structure, O doping can also tune the local atomic arrangement, in which the structure distortion can be observed from TEM image and further testified by X‐ray absorption fine structure spectroscopy (XAFS). Furthermore, average recovery lifetime of photogenerated electrons in O‐doped ZnIn_2_S_4_ is increased by 1.53 times relative to pristine ZnIn_2_S_4_ nanosheets, confirmed by ultrafast transient absorption spectroscopy. Benefiting from these regulation, the O‐doped ZnIn_2_S_4_ nanosheets deliver an outstanding photocatalytic hydrogen evolution performance, reaching 2120 µmol h^−1^ g^−1^ under visible light, ≈4.5 times higher than pristine ZnIn_2_S_4_ nanosheets.

### 3.2 Vacancy Engineering

**Figure 4:**
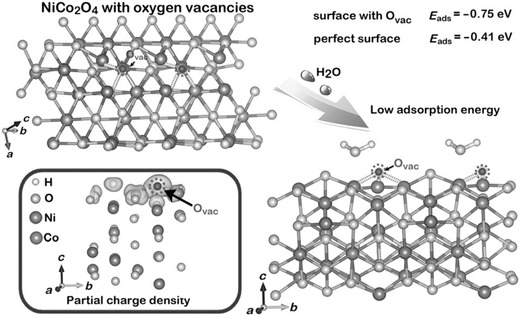
Schematic illustration of the adsorption of H_2_O molecules onto the spinel structure and the partial charge density of NiCo_2_O_4_ with oxygen vacancies. Reproduced with permission.^66^ Copyright 2015, Wiley‐VCH.

Vacancy engineering has been regarded as a powerful strategy to tune the surface atomic microstructure to affect the interfacial catalytic processes or tune the electronic structure to optimize the carrier concentration and charge transport.^65^ By virtue of the construction of various surface vacancies, the photoelectric catalysis properties can be thus optimized. For example, NiCo_2_O_4_ ultrathin nanosheets with different oxygen vacancy concentrations can be controlled prepared through a phase transformation strategy under diverse atmosphere and employed for OER.^66^ Apart from the number of active sites, the reactivity of active sites is also quite important to determine the OER performance, in which the interaction between the O_2_/H_2_O and catalyst surface is rather associated with the reactivity. According to the density functional theory (DFT) calculation, the oxygen vacancy is found able to decrease the hindrance for the adsorption of H_2_O on the surface of NiCo_2_O_4_ (**Figure** **4**). Co^3+^ center is more active toward the adsorption of H_2_O near an oxygen vacancy in the surface to achieve a coordination number of six, enabling the defective NiCo_2_O_4_ with a lower adsorption energy (−0.75 eV) for H_2_O relative to perfect NiCo_2_O_4_ (−0.41 eV). The greatly decreased H_2_O adsorption energy at the oxygen vacancy sites make the H_2_O easier to involve in the OER process, and result in significantly improved electrocatalytic oxygen evolution performance. The defective NiCo_2_O_4_ nanosheets can deliver an overpotential (η) of 0.32 V to achieve a current density of 10 mA cm^−2^ in 1 m KOH solutions as well as a large current density of 285 mA cm^−2^ at 0.8 V.

Apart from the optimizing of surface adsorption, the vacancy engineering can also tune the electronic structure and carrier transport. Jiao et al. synthetized defective one‐unit‐cell ZnIn_2_S_4_ with tunable zinc vacancy (V_Zn_) concentrations.^18^ The distinct V_Zn_ concentrations can be directly observed by aberration‐corrected scanning transmission electron microscopy and further certified by positron annihilation spectrometry and electron spin resonance. According to the DFT calculations, the presence of zinc vacancy could lead to significantly increased charge density at the nearby sulfur atoms, which reveals the electrons in V_Zn_‐rich one‐unit‐cell ZnIn_2_S_4_ can be easily excited to the CB and favors carriers' separation. The zinc vacancies can also work as electron capture centers to form trap states, so as the recombination of electron–hole pairs can be effective restrained, as verified by surface photovoltage spectroscopy and ultrafast transient absorption spectroscopy. Benefiting from these advantages, the V_Zn_‐rich one‐unit‐cell ZnIn_2_S_4_ layers display a 3.6 times higher photocatalytic activity toward CO_2_ reduction relative to V_Zn_‐poor ZnIn_2_S_4_, with a CO formation rate up to 33.2 µmol g^−1^ h^−1^. Moreover, no obvious inactivation or structure variation can be observed for V_Zn_‐rich one‐unit‐cell ZnIn_2_S_4_ layers after 24 h photocatalytic reaction, making these materials to be one of the most stable photocatalysts for photocatalytic CO_2_ reduction to yield CO.

In addition to monovacancy, cooperation of multivacancies may further give an unexpected activity promotion role. For instance, water‐plasma‐enabled exfoliation was employed to prepare ultrathin CoFe LDHs nanosheets with multivacancies.^49^ The exfoliation of pristine CoFe LDHs was realized through the destruction of electrostatic interactions between the host metal layers by water plasma treatment. At the same time, the etching effect of plasma can create multivacancies (including O, Co, and Fe vacancies) in the prepared ultrathin nanosheets. It is astonishing that the ultrathin CoFe LDHs nanosheets with multivacancies exhibit a ultralow overpotential of only 232 mV at 10 mA cm^−2^ for OER in 1 m KOH and possesses a Tafel slope of 36 mV dec^−1^.

### 3.3 Pores Construction

Building pores on the 2D planes can provide several advantages toward the photo, electrocatalysis processes. First of all, the formed pores can build abundant coordination‐unsaturated active atoms with dangling bonds, serve as edge active sites. For another, the pores on the 2D plane can also shorten the charge diffusion distance since the charge will transport along the 2D plane to arrive the active sites. Moreover, the abundant pores can serve as permeable channels for vertical ion penetration and favors the gas product release, thus allowing more subsurface materials available to be catalytically active. As a result, it is desirable to construct pores on the ultrathin 2D planes to further boost the catalytic performance. Recently, Xie et al. introduced numerous nanopores into β‐Ni(OH)_2_ nanosheets through 2D‐confined etching‐intralayered Ostwald ripening process.^44^ Depend on prefabricated Ni‐Al LDH ultrathin nanosheets, the strong alkaline solution was employed to etching the nanosheets and extract the Al atoms and a few Ni atoms surrounded by Al. Subsequently, Ostwald ripening confined by the highly anisotropic 2D structure of β‐Ni(OH)_2_ happens to form a thermodynamically more stably highly porous β‐Ni(OH)_2_ nanostructure (named as β‐Ni(OH)_2_ ultrathin nanomeshes). Benefiting from the unique porous structure, β‐Ni(OH)_2_ ultrathin nanomeshes display remarkable OER performance with a low overpotential of 236 mV to drive a 20 mA cm^−2^ current. The β‐Ni(OH)_2_ ultrathin nanomeshes can acquire a TOF of 7.82 × 10^−2^ s^−1^ under overpotential of 350 mV, which is 59.4 times higher than that of β‐Ni(OH)_2_ ultrathin nanosheets. Similar to the electrocatalytic process, the pores construction strategy also works in photocatalytic reactions. Through ethylene glycol etching process, several atoms in the basic plane of BiOCl ultrathin nanosheets are lost to form pores.^26^ Due to the energy band structure diversity between (001) and (110) facets, the photogenerated electrons will transfer along (001) direction, while the holes will migrate along the basic plane to the (110) facets. The constructed pores in the basic plane can shorten the migration distance of holes and thus promote the charge separation efficiency. Moreover, the coordination‐unsaturated active atoms along the pores afford an outstanding chemical environment for promoting chemisorption of H_2_O, increasing oxygen evolution kinetics. As a consequence, the pores‐rich BiOCl nanosheets exhibit an improved photocatalytic oxygen evolution activity than BiOCl ultrathin nanosheets.

### 3.4 Surface Modification and Heterojunction

**Figure 5:**
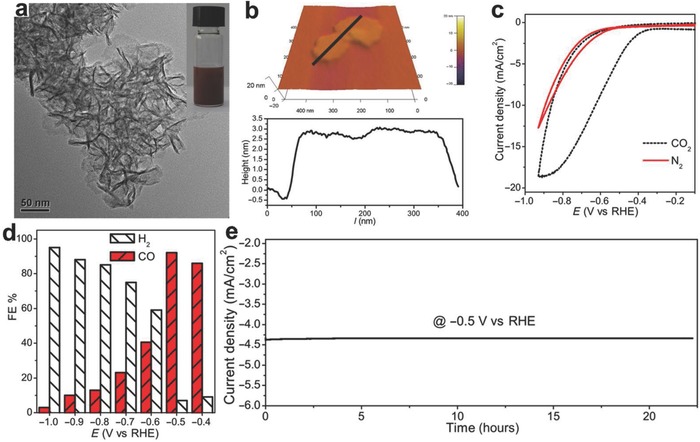
a) Low‐magnification TEM image. Inset: photograph of a dispersion of the nanosheets in ethanol. b) AFM image (top) and the height profile (bottom) of the nanosheets. c) Normalized polarization curves in N_2_‐saturated and CO_2_‐saturated 0.5 m NaHCO_3_ aqueous solution by the electrode surface area of electrocatalysts. The scan rate was 10 mV s^−1^. d) FEs of CO and H_2_ at different potentials. e) Chronoamperometric current at −0.5 V versus RHE. The loading of the Cu/Ni(OH)_2_ nanosheets on carbon paper was 0.5 mg cm^−2^. Reproduced with permission.^69^ Copyright 2017, the American Association for the Advancement of Science.

Surface modification and heterojunction are also considered as effective strategies to optimize the catalytic performance, not only for activity but also for stability.^67^, ^68^ Zheng's group prepared the 2.8 nm thick Cu/Ni(OH)_2_ nanosheets, with a Cu(0)‐enriched surface and surface‐stabilized by formate (**Figure** **5**).^69^ The samples are prepared by substituting the Ni^2+^ in beforehand synthetized Ni(OH)_2_ nanosheets with Cu^2+^. Subsequently, the Cu^2+^ are reduced to Cu_2_O and eventually to Cu(0) with the help of sodium formate to form Cu/Ni(OH)_2_ ultrathin nanosheets. The as‐synthesized Cu/Ni(OH)_2_ nanosheets display an outstanding catalytic performance and stability in the selective electroreduction of CO_2_ into CO. The Cu/Ni(OH)_2_ nanosheets displayed a current density of 4.3 mA cm^−2^ at overpotential (η) of 0.39 V, much higher than that of Ni(OH)_2_ nanosheets. It is well known that the Cu(0) nanomaterials are easily oxidized in air. The excellent electrocatalytic stability of the Cu/Ni(OH)_2_ nanosheets was derived from the surface formates modification to stabilize the metallic nature of Cu and Ni(OH)_2_ as support for preventing Cu nanosheets from being sintered.

**Figure 6:**
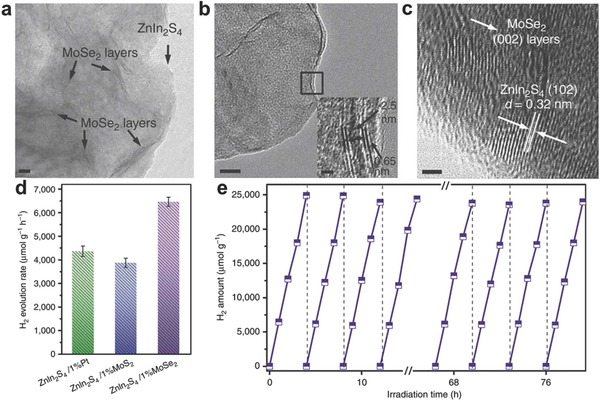
a,b) TEM and c) HRTEM images of hetero‐layered ZnIn_2_S_4_/MoSe_2_. Scale bar, a, b) 10 nm and c) 2 nm. d) Comparison of photocatalytic H_2_ evolution activities over ZnIn_2_S_4_/1%Pt, ZnIn_2_S_4_/1%MoS_2_, and ZnIn_2_S_4_/1%MoSe_2_. e) Recycling photoactivity test of ZnIn_2_S_4_/1%MoSe_2_. Reproduced with permission.^20^ Copyright 2017, Nature Publishing Group.

With respect to photocatalysis process, construction of 2D–2D ultrathin heterojunction have been demonstrated to be effective strategy to achieve high photocatalytic activity.^29^, ^32^, ^70^ The formed stacking interface with sufficient and tight contaction favors the efficient charge transfer across the interface and ensure the effective charge separation. Yang et al. developed a scalable self‐surface charge exfoliation and electrostatic coupling strategy to prepare ultrathin 2D hetero‐layered metal chalcogenides.^20^ Take the ZnIn_2_S_4_/MoSe_2_ as example, single‐unit‐cell ZnIn_2_S_4_ layers can be obtained via a low‐temperature refluxing and followed by a water‐assisted exfoliation process (**Figure** **6**). Since the ultrathin ZnIn_2_S_4_ is negatively charged, the positively charged MoSe_2_ can couple with ZnIn_2_S_4_ to form a hetero‐layered ZnIn_2_S_4_/MoSe_2_ by strong electrostatic attraction. The efficient charge transfer and intimate contact interface can be achieved in hetero‐layered ZnIn_2_S_4_/MoSe_2_, endowing an excellent visible light photocatalytic H_2_ production behavior, up to 6454 µmol g^−1^ h^−1^. This H_2_ production rate is higher than that of single‐unit‐cell ZnIn_2_S_4_ layers (1748 µmol g^−1^ h^−1^), ZnIn_2_S_4_/1%Pt (4353 µmol g^−1^ h^−1^) and ZnIn_2_S_4_/1%MoS_2_ (3860 µmol g^−1^ h^−1^). More importantly, optimal ZnIn_2_S_4_/MoSe_2_ shows excellent stability, with negligible photoactivity loss can be seen after 20 consecutive cycles in 80 h. Unfortunately, the sacrificial electron donor (lactic acid) was still required during the H_2_ production process, veritable water splitting with production of hydrogen and oxygen in stoichiometric ratio 2:1 cannot be obtained in this system.

## 4 Energy Catalytic Applications

### 4.1 Oxygen Evolution Reaction

Water splitting and CO_2_ reduction driven by the solar energy or renewably generated electricity has been considered as effective pathway to address the increasingly energy shortage issue. However, the catalytic efficiency is still far from ideal and one of the significant factors that limit the practical utilization is the sluggish reaction kinetics of oxygen evolution half‐reaction.^71^, ^72^ Studies found that coupling effect between ultrathin structure construction and multinary component tuning can create outstanding OER performance.^73^ Hu's group demonstrated that the liquid phase exfoliation can be applied in layered double hydroxides (such as CoCo, NiCo, and NiFe LDHs) to acquire single‐layer nanosheets.^46^ The exfoliated nanosheets can display a significantly higher OER activity than corresponding bulk counterpart. Further studies found that the catalytic activity has the order of NiFe>NiCo>CoCo for exfoliated nanosheets and NiFe LDHs single‐layer nanosheets possess an optimal performance with at an overpotential of 300 mV to acquire a 10 mA cm^−2^ current density. This result undoubtedly indicates the thickness controlling and component tuning are important strategies to couple‐promote the OER behavior.

**Figure 7:**
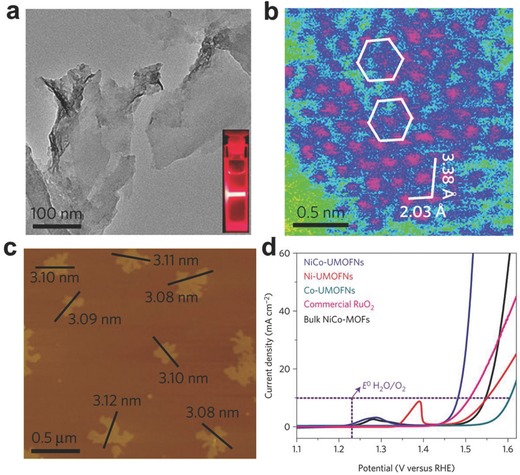
a) TEM image of NiCo‐UMOFNs. b) HAADF‐STEM image of the NiCo‐UMOFNs. The pink color represents metal atoms, blue is for light elements (carbon and oxygen), and green is for background. c) AFM image of NiCo‐UMOFNs. d) Polarization curves of samples in O_2_‐saturated 1 m KOH solution at a scan rate of 5 mV s^−1^. Reproduced with permission.^54^ Copyright 2016, Nature Publishing Group.

Tang's group synthesized NiCo bimetal‐organic framework nanosheets (NiCo‐UMOFNs) with a uniform thickness of ∼3.1 nm (**Figure** **7**).^54^ Benefiting from the advantage of high‐angle annular dark‐field scanning TEM (HAADF‐STEM) imaging under the low‐voltage mode, the exact metal atoms arrangement on the (200) planes of NiCo‐UMOFNs can be directly observed. The as‐prepared NiCo‐UMOFNs can deliver an overpotential of only 189 mV to achieve a current density of 10 mA cm^−2^ for electrocatalytic OER when loaded on copper foam. This is the lowest electrocatalytic OER overpotential to give a current density of 10 mA cm^−2^ over atomically thin 2D multinary nanosheets so far. As evidenced by X‐ray absorption spectra and theory (DFT) calculations, the coordinatively unsaturated surface atoms in the ultrathin MOF sheets are regarded as open sites for adsorption and are dominating active centers with a synergistic effect between Ni and Co to promote the OER activity.

### 4.2 Oxygen Reduction Reaction

Apart from OER, oxygen reduction reaction (ORR) is also an important energy‐related reaction in metal–air batteries and fuel cells. In practice, the ORR kinetics at the cathode dominate the overall efficiency of low‐temperature fuel cells. However, due to the features of scarcity, high cost and easy deactivation by CO of the widely applied Pt electrocatalyst, it is desirable to further develop outstanding alternative. Recent studies found that atomically thin 2D multinary nanosheets may be one kind of effective alternative.^74^ For instance, Jing et al. prepared FeNiS_2_ ultrathin nanosheets with the thickness of 2–3 nm via a facile colloidal method.^75^ The obtained FeNiS_2_ ultrathin nanosheets can deliver an onset potential of 0.78 V (vs reversible hydrogen electrode (RHE)), a Tafel slope of 107 mV decade^−1^ under neutral conditions, much superior than the corresponding binary FeS and Ni_9_S_8_ ultrathin nanosheets. At the same time, a high selectivity with electron transfer number of 3.92 and H_2_O_2_% of 3.6% at 0.3 V can be achieved for FeNiS_2_ ultrathin nanosheets. Although the onset potential and diffusion‐limiting current density of FeNiS_2_ ultrathin nanosheets is still some inferior than commercial Pt/C, the stability is much superior than Pt/C.

### 4.3 Hydrogen Evolution Reaction

Owning to the favorable energy density and environmental friendliness, hydrogen is regarded as one of the most promising candidates to replace fossil fuel. Since the steam‐reforming reaction is the dominated approach to produce hydrogen in industries currently, a large amount of CO_2_ will be discharged as waste, which is unfavorable for environment. It is desirable to develop sustainable routes such as electrocatalytic or photocatalytic processes to produce hydrogen.^76^, ^77^ In acidic solutions, the HER reaction starts with the adsorption of proton onto the catalyst (M) surface (Volmer step, H_3_O^+^ + *e* ^−^ + M ↔ MH_ads_ + H_2_O), followed by either reaction of adsorbed hydrogen (H_ads_) with hydrated proton (Heyrovsky step, MH_ads_ + H_3_O^+^ + *e* ^−^ ↔ H_2_ + H_2_O + M) or combination of two H_ads_ (Tafel step, 2[MH_ads_] ↔ H_2_ + 2M) to generate hydrogen gas. In this field, atomically thin 2D multinary nanosheets also display giant potential to deliver outstanding behavior. Aiming at either NiPS_3_ or FePS_3_ is not a good HER electrocatalyst, Song et al. employed appropriate amount of Fe into NiPS_3_ to tune the electronic structure and improve electrical conductivity, and eventually optimize the HER performance.^78^ According to the DFT calculation, the hydrogen adsorption free energy Δ*G*_H_ of different sites was calculated on the both basal surface and edges. The surface is founded to be inactive for the HER and the Δ*G*_H_ value on the S site in 10% Fe doped NiPS_3_ can be reduced to merely 0.06 eV, suggesting the possible excellent HER performance. At the same time, alloying Fe into NiPS_3_ can effective improve the electrical conductivity. The optimized Ni_0.9_Fe_0.1_PS_3_ nanosheets with 4 nm thick can gain a current density of −10 mA cm^−2^ with an overpotential of 72 mV and a Tafel slope of 73 mV dec^−1^, fairly superior than that of NiPS_3_ and FePS_3_.

**Figure 8:**
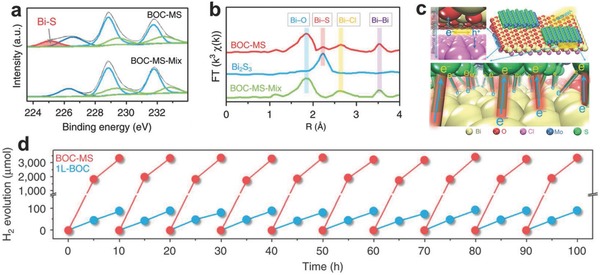
Comparison of a) XPS spectra and b) Bi L‐edge EXAFS of BOC‐MS and BOC‐MS‐Mix, identifying the formation of Bi—S bonds. (c) Schematic illustration of electron–hole separation within Bi_12_O_17_Cl_2_ and the interfacial electron transfer from Bi_12_O_17_Cl_2_ to MoS_2_ along the Bi—S bonds. (d) Cycling tests of photocatalytic hydrogen evolution over BOC‐MS and Bi_12_O_17_Cl_2_ single layer. Reproduced with permission.^29^ Copyright 2016, Nature Publishing Group.

Previous studies found that BiOCl ultrathin nanosheets cannot display good photocatalytic hydrogen evolution activity due to the narrow photoabsorption range, high charge recombination rate and lack of hydrogen evolution sites.^27^, ^28^ To overcome these weakness, Li et al. employed bismuth‐rich strategy to tune the electronic structure, and coupling single‐layer MoS_2_ to promote charge separation and supply hydrogen evolution sites (**Figure** **8**).^29^ The Bi_12_O_17_Cl_2_ single layer serve as photocarrier generator to produce electron–hole pairs, then electron across to interface via Bi—S bonds to MoS_2_ layer, resulting in a ultralong carrier lifetime of 3446 ns. Consequently, the MoS_2_/Bi_12_O_17_Cl_2_ bilayer junctions exhibit an advanced visible‐light hydrogen evolution rate of 33 mmol h^−1^ g^−1^, with quantum yield of 36% at 420 nm. This is the highest formation rate for H_2_ so far under visible light illumination over atomically thin 2D multinary nanosheets.

### 4.4 Carbon Dioxide Reduction

**Figure 9:**
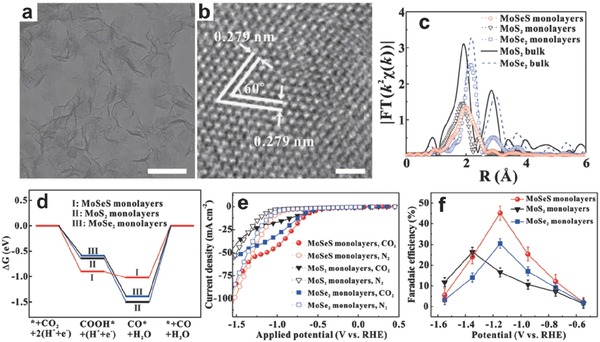
a) TEM image, scale bar: 100 nm; b) HRTEM image, scale bar: 1 nm of MoSeS alloy monolayers. c) Fourier transforms FT(*k* ^2^χ(*k*)) for the samples. d) Calculated free energy diagrams for CO_2_ electroreduction to CO. e) Linear sweep voltammetric curves in the CO_2_ saturated solution. f) Faradaic efficiencies (FE) for CO at different applied potentials for each 2.5 h. Reproduced with permission.^23^ Copyright 2017, Wiley‐VCH.

Due to the excessive use of fossil fuel, greatly increased CO_2_ concentration in atmosphere has bring about raised global mean temperature and severe climatic hazards. Converting CO_2_ into value‐added carbon‐based fuel or chemicals to build a sustainable recycling system has been regarded as an effectively pathway to simultaneously relieve energy crisis and environmental issues.^79^, ^80^ The first step for the CO_2_ reduction is adsorbing the CO_2_ on the catalyst surface and activate CO_2_ via single electron to form CO_2_^·−^ intermediate. Due to the large reorganizational energy between the linear molecule of CO_2_ and bent radical anion of CO_2_^·−^, this process with a very negative equilibrium potential (−1.9 V versus NHE) has been considered as the rate‐limiting step for the CO_2_ reduction. To bypass the formation of CO_2_^·−^, proton‐assisted multiple‐electron transfer processes with lower energetic costs are always proceed. Different products such as HCOOH, CO, HCHO, CH_3_OH, and CH_4_ can be obtained via different numbers of transfer electrons, 2e^−^, 2e^−^, 4 e^−^, 6 e^−^, and 8 e^−^. Corresponding, the redox potentials can be reduced to −0.61, −0.53, −0.48, −0.38, and −0.24 V, versus NHE at pH = 7, respectively. CO_2_ reduction through electrocatalytic or photocatalytic processes over atomically thin 2D multinary nanosheets may be an effective approach.^81^, ^82^ Recently, Xu et al. prepared MoSeS alloy monolayer with the thickness of 0.74 nm via a liquid–liquid interface‐mediated strategy (**Figure** **9**).^23^ By virtue of XAFS measurements, the distinct local atomic arrangements of MoSeS alloy monolayer was found to suffer from significant variation relative to the MoS_2_ and MoSe_2_ monolayers, with shortened Mo—S bond length and lengthened Mo—Se bond. The increased DOS near the CB edge in MoSeS alloy monolayer can be observed, which could enable an increased electrical conductivity. Furthermore, the off‐center charge density of Mo atom can help stabilize COOH* intermediate and also facilitates the CO desorption. As a consequence, compared with the MoS_2_ monolayers and MoSe_2_ monolayers, the MoSeS alloy monolayers display improved electrocatalytic activity toward CO_2_ reduction, which can acquire a Faradaic efficiency up to 45.2% for CO production at −1.15 V versus RHE.

Hou et al. engineered phosphate and oxygen‐vacancy to simultaneously optimize the photocatalytic CO_2_ reduction performance of Bi_2_WO_6_ atomic layers.^41^ The engineered oxygen‐vacancy can extend the photoabsorption range to even the whole spectral region. At the same time, the phosphate modification and engineered oxygen‐vacancy can greatly promote the charge separation efficiency, as evidenced by electrochemical impedance spectra, steady‐state PL spectra and time‐resolved PL decay curves. The enhanced light absorption and charge transport behavior endow the V_o_‐PO_4_‐Bi_2_WO_6_ with higher methanol formation rate of 157 µmol g^−1^ h^−1^ under 300 W Xe lamp with a standard AM 1.5 G filter, over 2 times higher than Bi_2_WO_6_ atomic layers.

### 4.5 Nitrogen Fixation

**Figure 10:**
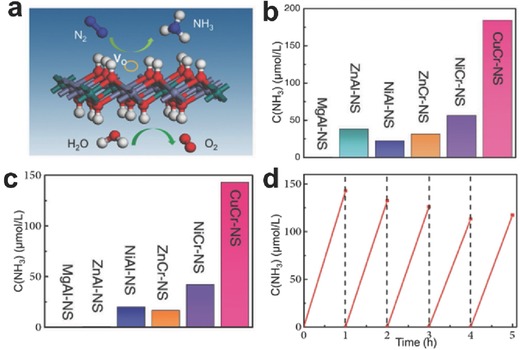
a) Schematic illustrating the photocatalytic N_2_ fixation process. Yield of NH_3_ over a 1 h test period for different LDH photocatalysts under b) UV–vis illumination and c) visible‐light illumination (λ > 400 nm, PLS‐SXE300D) with water as the proton source. d) Catalyst cycling tests for CuCr‐NS under N_2_ in the presence of water under visible‐light illumination. Reproduced with permission.^53^ Copyright 2017, Wiley‐VCH.

Currently, NH_3_ production through highly energy‐consuming Haber–Bosch process consumes 2% fossil fuels in its global total supply. Developing alternative nitrogen fixation technology with sustainable energy input can significantly reduce the use of fossil fuels as energy source and decrease the carbon emissions.^83^ With respect to the competitive photocatalytic nitrogen fixation technology, the inexhaustible light energy can be stored into chemical energy and can further be converted to electric energy via fuel cell.^84^ The nitrogen fixation to produce NH_3_ is thermodynamically accessible: N_2_(g) + 3H_2_(g) → 2NH_3_(g), Δ*H*_298K_ = −92.2 kJ mol^−1^. However, it cannot occur spontaneously under ambient conditions owing to the strong triple bond energy of N_2_ is as high as 962 kJ mol^−1^ and even the cleavage energy of the first bond in nitrogen can arrive 410 kJ mol^−1^. To build strong binding force of N_2_ with the surfaces of catalysts and facilitate the activation, surfaces with strong electron donors and abundant catalytic activation centers to facilitate charge transfer from the catalyst to N_2_ are highly desired. Recently, Zhao et al. demonstrated that ultrathin LDH photocatalysts with distorted structure induced by oxygen vacancies can display remarkable photocatalytic activity by reducing N_2_ to NH_3_ (**Figure** **10**).^53^ Through the metal ion regulation, the CuCr LDH ultrathin nanosheets (CuCr‐NS) was found to possess optimal photocatalytic activity, in which the NH_3_ concentration can reach 184.8 µmol L^−1^ or 142.9 µmol L^−1^ under UV–vis or visible light irradiation, respectively. By virtue of XAFS spectra and positron annihilation spectrometry, abundant surface oxygen defects and compressive bonding can be found to exist in LDH nanosheets owning to the atomic‐thin thickness. Furthermore, Cu(II) ions induced Jahn–Teller distortions making CuCr‐NS with more surface defects relative to ZnCr‐NS. Benefiting from the defect structure, the electronic structure and photoexcited charge‐transfer properties can be tuned as well as the dinitrogen can be better adsorbed and activated. Eventually, the CuCr‐NS with structural distortions and compressive strain deliver a good yield of NH_3_ under photocatalytic process at mild reaction conditions.

## 5 Summary and Outlook

Atomically thin 2D multinary nanosheets have been reviewed with classification, synthesis, performance optimization, and energy‐related photo, electrocatalytic applications, which may have a significant impact to solve future energy and environment issues. Moreover, the strategies to tune the microstructure, electronic structure, chemical and optical properties so as to boost the catalytic performance have been summarized, such as alloying, doping, vacancy engineering, pores construction, surface modification, and heterojunction. Generally, the photocatalysts possess relative larger bandgap than electrocatalysts but should not be too large (usually 2–4 eV), while the electrocatalysts usually have the bandgap less than 2 eV. Despite the significant progress, it still remains several unexplored aspects yet to be investigated, which can be considered as opportunities in future research.

First of all, it is hard to prepare the atomically thin 2D multinary nanosheets in large‐scale and this may be a major challenge to restrict the industry applications. A mass of the prepared samples lacks the long‐term stability and durability. It is desirable to develop effective method to solve the large‐scale production and store issues of atomically thin 2D multinary nanosheets. Next, although the multinary nanosheets show some advances than the corresponding monocomponent or binary nanosheets, the component adjustment mainly depends on the spontaneous process of materials. Further tuning the ratio of various components to optimize the catalytic performance is highly desired since the metastable phase may have higher activation ability to facilitate the interfacial reactions. Moreover, in general, the reaction mechanism of hydrogen generation is more clear than CO_2_ reduction. Since the multiple surface adsorption pattern and various reaction products, the CO_2_ adsorption, activation, multielectron transfer, and desorption processes are unclear in some systems. In the hydrogen production process, the Gibbs free energy for hydrogen adsorption (Δ*G*_H*_) has been regarded as a good descriptor for the catalytic activity. The near‐zero Δ*G*_H*_ of Pt enables it to be powerful HER catalysts, while the single metal materials are usually not so excellent toward CO_2_ reduction. It is desirable to explore appropriate descriptor to evaluate CO_2_ reduction activities as well as develop 2D multinary nanosheets to boost the performances. The accurate reaction mechanism and catalytically active sites for different processes are still not clear in several systems. Advanced in situ characterization techniques are highly desired to be developed so as to have deep insight into the reactive intermediates, reaction pathways, and dynamics and build clearly structure–activity relationships in different reaction systems. The synergistic utilization of theory and advanced characterizations will help to determine the different types of catalytic active sites and contribute to increasing the number or boost the inherent activity of each active site. The exact guidance will further endow the novel atomically thin 2D multinary nanosheets with tunable and synergetic catalytic active sites toward arduous catalytic reactions.

## Conflict of Interest

The authors declare no conflict of interest.
